# Left-side vs. right-side hepatectomy for hilar cholangiocarcinoma: a meta-analysis

**DOI:** 10.1186/s12957-021-02213-6

**Published:** 2021-04-10

**Authors:** Wenxuan Wu, Qiyang Cheng, Junru Chen, Diyu Chen, Xiaode Feng, Jian Wu

**Affiliations:** 1 Division of Hepatobiliary and Pancreatic Surgery, Department of Surgery, First Affiliated Hospital, Zhejiang University School of Medicine, Hangzhou, 310003 Zhejiang Province China; 2 Key Laboratory of Combined Multi-organ Transplantation, Ministry of Public Health, First Affiliated Hospital, Zhejiang University School of Medicine, Hangzhou, 310003 China; 3 Key Laboratory of Organ Transplantation, Research Center for Diagnosis and Treatment of Hepatobiliary Diseases, Hangzhou, 310003 Zhejiang Province China

**Keywords:** Hilar cholangiocarcinoma, Perihilar cholangiocarcinoma, Left-side hepatectomy, Right-side hepatectomy, Meta-analysis

## Abstract

**Goals:**

We aim to draw a conclusion which type of hepatectomy could be the priority for hilar cholangiocarcinoma patients.

**Background:**

Surgery is established as only potentially curative treatment for hilar cholangiocarcinoma. However, whether hepatectomy should be preferred to the left-side hepatectomy, which includes left hemihepatectomy, extended left hemihepatectomy, and left trisectionectomy, or right-side hepatectomy, which represents right hemihepatectomy, extended right hemihepatectomy, and right trisectionectomy, is debated. In this meta-analysis, we evaluated and compared the efficacy and safety of left-side hepatectomy and right-side hepatectomy in patients with hilar cholangiocarcinoma.

**Study:**

We systematically retrieved the MEDLINE, PubMed, and Cochrane library and related bibliography up to February 2020. The primary outcome is overall survival, and the secondary outcome includes 1-, 3-, and 5-year survival rates, morbidity, mortality, R0 resection rate, and operation time. Based on heterogeneity, fixed-effects model or random-effects models were established through meta-analysis.

**Results:**

Eleven studies (11 cohort studies, totally 1031 patients) were involved in this study. The overall survival of patients who underwent left-side hepatectomy was comparable to that of patients who underwent right-side hepatectomy (hazard ratio, 1.27 [95% confidence interval, 0.98–1.63]). And there was no significant difference observed in 1-year (relative risk, 1.01 [95% CI, 0.89–1.15]), 3-year (relative risk, 0.94 [95% confidence interval, 0.80–1.11]), and 5-year survival (relative risk, 0.82 [95% confidence interval, 0.67–1.01]) rates between the left-side hepatectomy group and the right-side hepatectomy group. Comparing with the right-side hepatectomy cluster, the hilar cholangiocarcinoma patients in the left-side hepatectomy cluster presented better overall postoperative morbidity (relative risk, 0.82 [95% confidence interval, 0.71–0.96]) and major postoperative morbidity (relative risk, 0.73 [95% confidence interval, 0.56–0.95]). The post-hepatectomy liver failure rate (relative risk, 0.22 [95% confidence interval, 0.09–0.56]) and procedure-related mortality (relative risk, 0.41 [95% confidence interval, 0.23–0.70]) in the left-side hepatectomy group were better than those of the right-side hepatectomy group. Besides, the R0 resection rate was similar between the left-side hepatectomy group and the right-side hepatectomy group (relative risk, 0.95 [95% confidence interval, 0.87–1.03]). And the operation time for the left-side hepatectomy was significantly longer than that for the right-side hepatectomy (mean difference, 38.68 [95% confidence interval, 7.41–69.95]).

**Conclusion:**

Through meta-analysis, we explored the comparable long-term outcomes and better short-term outcomes in the left-side hepatectomy group as is compared to the right-side hepatectomy group of hilar cholangiocarcinoma patients. In this study, the evidence obtained might indicate that the choice of left-side hepatectomy or right-side hepatectomy depends on the site of hilar cholangiocarcinoma in every patient.

**Supplementary Information:**

The online version contains supplementary material available at 10.1186/s12957-021-02213-6.

## Introduction

Hilar cholangiocarcinoma (HCCA), a type of cholangiocarcinoma, is classified based on the anatomical location and is located in the area between the second-degree bile ducts and the insertion of the cystic duct into the common bile duct [1]. The prognosis of HCCA patients is poor. Radical surgery with negative margins (R0) is the only potentially curative treatment for this disease. However, frequent metastasis and recurrence remain the major obstacles for the prognosis of HCCA patients who underwent surgical resection (a 1-year survival rate of 80% and a 5-year survival rate of 39%) [2, 3]. Recently, it is considered to be the standard surgical procedure of HCCA, which includes bile duct resection combined with major hepatectomy, caudate lobe resection, lymph node dissection, and vascular resection when necessary, resulting in improved R0 resection rate and long-term survival [4–8].

Up to now, right-side hepatectomy (RH) is recognized as an accepted option for major liver resection in HCCA treatment [9, 10]. Tumor location is a major factor in operation methods selection for HCCA, and the following factors also should be considered: (1) Length of hepatic duct: the extrahepatic portion of left hepatic duct is longer than the right one. (2) Oncological characteristic: due to the vertical spread characteristic of HCCA, the right hepatic artery is susceptible to be invaded. Moreover, the right hepatic artery usually travels behind the proximal bile duct near the hepatic hilum, making RH more advantageous in terms of radicality. (3) The anatomical structure on the right side of the hepatic hilum is complicated, with many anatomical variations; (4) it is easier to complete caudate lobectomy [10–13]. However, RH is confirmed to be the risk of future liver remnant (FLR) deficiency and even postoperative liver failure (PHLF). Although preoperative biliary drainage and portal vein embolization (PVE) were utilized into the HCCA preoperative management, it is still unclear whether these measurements could improve in postoperative morbidity and mortality [14, 15].

Whereas left-side hepatectomy (LH) is more complicated and sometimes arterial reconstruction is needed during the operation [16], it is still an essential option for the HCCA located in the left liver [17, 18]. Generally, because of the anatomical structure, the patients who underwent LH possess more FLR volume, which means it could take patients from less PHLF risk.

Many studies reported that RH can achieve better long-term survival resulted from higher R0 resection rate [13, 16]. Nevertheless, Govil et al.’s [19] research reveals that LH is comparable to RH in long-term survival. Due to the rarity of HCCA and the small number of cases, the comparison between the effects of LH and RH remains unknown.

The aim of this meta-analysis is to conduct a statistical evaluation based on the existing studies, to clarify the long-term outcome of the LH and RH of HCCA, and to compare the differences of short-term outcome, R0 resection rate, and operation time, in order to provide evidence for clinical application.

## Materials and methods

### Search strategy

This meta-analysis followed the Preferred Reporting Items for Systematic Review and Meta-Analyses (PRISMA) statement [20] and the Meta-analysis of Observational Studies in Epidemiology (MOOSE) guidelines [21]. A comprehensive systematic search was performed on PubMed, EMBASE, and Cochrane Library through to February 3, 2020. The search strategy was to combine keywords including “hilar cholangiocarcinoma,” “Klatskin tumor,” “left-side hepatectomy,” and “right-side hepatectomy” into various combinations. To identify more relevant literature, a manual search was performed on references of all included literature. Restrictions were not placed on any point of the search. In addition, the search process was completed independently by two authors (Wenxuan Wu and Qiyang Cheng), and the disagreement reached consensus through discussion. Our protocol was registered on the International Platform of Registered Systematic Review and Meta-Analysis Protocols INPLASY (https://inplasy.com/), and the registration number is INPLASY202130004. Moreover, our protocol is available with the DOI number (10.37766/inplasy2021.3.0004).

### Inclusion criteria and exclusion criteria

Two authors (Wenxuan Wu and Qiyang Cheng) independently screened the titles and abstracts of all literatures and reviewed further full texts if appropriate. Literature that reported the outcomes of left-side hepatectomy versus right-side hepatectomy in patients with HCCA and met the following criteria were included: (i) randomized controlled trials (RCTs), cohort studies, or case-control studies; (ii) adult patients with HCCA; (iii) language-free publication comparing left-side hepatectomy and right-side hepatectomy for HCCA; and (iv) include at least one of the following endpoints: overall survival (OS), 1-year survival rate, 3-year survival rate, 5-year survival rate, operating time, R0 resection rate, postoperative morbidity, PHLF, procedure-related mortality. Exclusion criteria include the following: (i) study design type without explicit accountability; (ii) patients with intrahepatic cholangiocarcinoma, distal cholangiocarcinoma, and gallbladder carcinoma; (iii) no controls; (iv) duplicates; (v) unable to extract valid outcome data from the literature; and (vi) conference, editorials, reviews, case reports, commentaries, letters, research involving animal experiments, cohorts with fewer than 10 cases, and when full text was not available.

### Data abstraction and quality assessment

For each literature included, data were extracted independently by two authors (Wenxuan Wu and Junru Chen) using a pre-made spreadsheet. The data to be extracted include (i) general information: first author, year of publication, and country; (ii) study characteristics: study design, study period, sample size, and duration of follow-up; (iii) patient and preoperative characteristics: gender, age, Bismuth classification, proportion of biliary drainage including percutaneous biliary drainage (PBD) and endoscopic biliary drainage (EBD) before surgery, and proportion of portal vein embolization (PVE); (iv) operative data: type of resection, operation time, R0 resection rate, and proportion of caudate lobectomy; (5) postoperative data: overall survival (OS), 1-year survival rate, 3-year survival rate, 5-year survival rate, postoperative morbidity, PHLF, and procedure-related mortality.

The primary endpoint of analysis was OS. OS was calculated from the time of surgery to the death or last contact. Postoperative morbidity includes overall morbidity and major morbidity (according to Clavien-Dindo classification, Dindo grades III–V). Procedure-related mortality was considered to include operative mortality, postoperative mortality, in-hospital mortality, and 90-day mortality. Hazard ratio (HR) is most appropriate for analyzing time-to-event outcomes. Given that only two literatures reported the values directly, this meta-analysis used the method of Parmar et al. [22] to extract data from the Kaplan-Meier curve and then used the Excel sheet published by Tierney et al. [23] to calculate HR.

Similarly, two authors (Wenxuan Wu and Junru Chen) independently assessed the quality of each literature. The Newcastle–Ottawa Quality Assessment Scale (NOS) [24] was used to assess quality of the cohort study. This tool includes three categories of selection, comparability, and outcome, with a maximum score of 9 stars and more than 6 stars are considered as high quality.

### Statistical analysis

To compare OS, we used HR and its 95% confidence interval (CI), and the other dichotomous data were calculated using relative risk (RR) and its 95% CI. Continuous data were presented as mean difference (MD) with 95% CI. Some literatures used the median and range to describe continuous data. In order to calculate uniformly, we used the formulas and tables provided by Luo et al. [25] and Wan et al. [26] to convert the data into mean and standard deviation (SD). Heterogeneity among the included studies was assessed using the *Q* test, and *P* < 0.1 was considered heterogeneous. The value of *I*^2^ is used to quantify the degree of heterogeneity, specifically, when the *I*^2^ values were 25%, 50%, and 75%, the corresponding heterogeneity is low, medium, and high [27]. Fixed-effects model was selected when there is no heterogeneity; otherwise, random-effects model was considered for pool data. Subgroup analyses were performed to assess the impact of region and year of publication on surgical outcome and survival, taking into account differences in treatment and surgical outcomes between eastern and western centers, as well as the ongoing development of modern surgical techniques. The cutoff point for subgroup analysis is the mean of the year of publication. Plotting a funnel plot and visually evaluating the symmetry of the funnel plot were done to see if there were publication biases. Begg’s test and Egger’s test were also conducted to explore potential publication bias, with a cutoff level of *P* < 0.05. Unless otherwise noted, two-sided *P* values < 0.05 were considered statistically significant. All statistical analyses for the meta-analysis were generated using STATA/MP software (version 14.0).

## Results

### Literature search

**Fig. 1:**
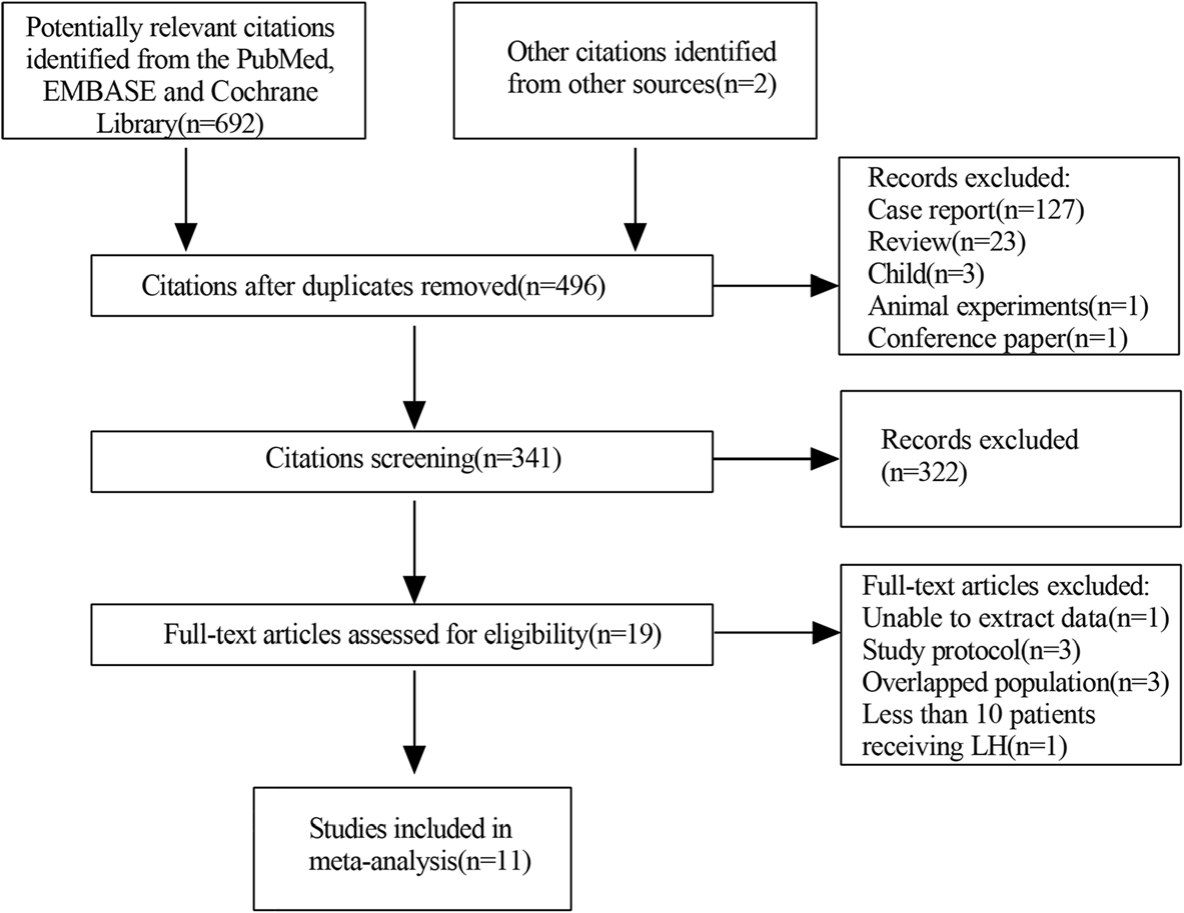
PRISMA flow diagram of study selection

Through a comprehensive search in PubMed, EMBASE, and Cochrane Library databases, a total of 694 citations were identified. Subsequently, after excluding 198 duplicate articles, our analysis removed 155 citations including case reports, reviews, conference papers, animal experiments, and research on children (Fig. 1). Based on screening the title and abstract, an additional 322 citations were excluded, and finally, 19 unique citations entered the full-text review. In order to retain the most recent and complete data, three studies based on the same population were eliminated after the further discussion by the two authors (Diyu Chen and Xiaode Feng). Eventually, 11 eligible cohort studies were included in this analysis [11, 15, 19, 28–35].

### Characteristics of included studies and assessment of methodological quality

The 11 eligible retrospective-prospective cohort studies were carried out between 2001 and 2020, including a total of 1031 patients. All studies were single-center studies, most of them (7/11) were performed in Asian populations (Japan, Korea, and India), and the others (4/11) were based on Western populations (Germany, Italy, and USA). Seven studies were published before 2014, and four after 2014. Nine cohorts involved in our study represent the usage of PVE, seven of which conducted PVE before RH, while two of which conducted it not only before RH but also before LH. As for caudate lobectomy, all patients underwent caudate lobectomy in six of eleven papers, a part of patients had been performed with caudate lobectomy in four studies, and the remaining one study described unclearly about this. The specific characteristics and data of the included studies were shown in the Table 1.

**Table 1:** Characteristics of the included studies

Study	Study design	Location/period	Follow-up (months^a^)	No. of patients (male, %)	Age, (years^a^)	No. of stage I/II/III/IV	No. of biliary drainage (EBD/PBD)	No. of PVE	Caudate lobectomy, %	Main findings
Bednarsch et al. [28]	Cohort study	Germany/ 2011–2016	28 (0–90)	LH: 36 (63.9)	67 ± 9	1/0/23/12	35 (27/8)	0	100	3- and 5-year OS rate, LH = 62%,30% vs. RH = 51%,46%; R0, LH = 69.4% vs. RH = 75.6%
				RH: 45 (68.9)	67 ± 11	2/6/23/14	46 (35/11)	37	100	
Govil et al. [19]	Cohort study	India/ 2009–2015	14 (3–64)	LH: 23 (NR)	58 (20–74)	0/0/28/8	8 (0/8)	0	NR	2-year OS rate, LH = 39%vs. RH = 44%, R0, NR
				RH: 13(NR)			6 (0/6)	0	NR	
Hong et al. [29]	Cohort study	Korea/ 2000–2018	NR	LH: 82 (68.3)	63.46 ± 10.38	5/6/43/28	60	2	100	1-, 3-, and 5-year OS rate, LH = 87.3%, 38.2%, 24.7% vs. RH = 77.2%, 41.4%, 26.8%; R0, LH = 75.6% vs. RH = 72.8%
				RH: 114 (66.7)	63.64 ± 8.72	4/13/75/22	93	45	100	
Jo et al. [15]	Cohort study	Korea/ 2010–2017	19 (1–97)	LH: 24 (62.5)	71 (53–83)	IV: 7	22(14/8)	0	100	1-, 3-, and 5-year OS rate, LH = 82.6%, 50.6%, 40.5% vs. RH = 69.3%, 48.5%, 37.7%; R0, LH = 75% vs. RH = 75.8%
				RH: 33 (66.6)	66 (42–79)	IV: 12	29(20/9)	6	100	
Lee et al. [30]	Cohort study	Korea/ 1995–2012	NR	LH: 35 (57.1)	61.0 ± 8.1	IIIb:35	23	0	94.3	1-, 3-, and 5-year OS rate, LH = 80%, 47%, 35% vs. RH = 85%, 47%, 33%; R0, LH = 85.7% vs. RH = 82.5%
				RH: 103 (66)	62.1 ± 9.2	IIIa: 103	71	24	86.4	
Otto et al. [31]	Cohort study	Germany/ 1998–2011	NR	LH: 68 (75)	64 (39–83)	0/0/35/33	NR	0	100	1- and 5-year OS rate, LH = 72%, 22% vs. RH = 73%, 29%; R0, LH = 72.1% vs. RH = 82.4%
				RH: 68 (66.2)	62 (44–82)	1/0/37/30	NR	4	100	
Ratti et al. [32]	Cohort study	Italy/ 2004–2014	23(3–98)	LH: 44 (68.1)	59 (36–79)	1/17/13/13	23(6/16)^b^	0	97.7	3- and 5-year OS rate, LH = 49.5%, 35.3% vs. RH = 53.2%, 42.8%; R0, LH = 61.4% vs. RH = 75.4%
				RH: 61 (50.8)	62 (41–82)	1/20/15/25	39(9/22)^b^	29	93.4	
Shimizu et al. [11]	Cohort study	Japan/ 1984–2008	NR	LH: 88 (69.3)	67.0 ± 8.9	IIIb: 88	NR	5	100	1-, 3-, and 5-year OS rate, NR; R0, LH = 63.6% vs. RH = 69.1%
				RH: 84 (56)	67.1 ± 8.0	IIIa +V: 84	NR	32	100	
Sugiura et al. [33]	Cohort study	Japan/ 2002–2013	NR	LH: 12 (91.7)	65 (58–84)	2/10/0/0	NR	0	100	3- and 5-year OS rate, LH = 66.7%, 41.7% vs. RH = 70.8%, 49%; R0, NR
				RH: 24 (75)	68 (37–81)	8/16/0/0	NR	24	100	
Konstadoulakis et al. [34]	Cohort study	USA/ 1988–2006	38 ± 30.4	LH: 29	NR	NR	NR	0	77.6	1-, 3-, and 5-year OS rate, LH = 66.7%, 33.3%, 21.7% vs. RH = 85%, 63.2%, 50%; R0, NR
				RH: 20	NR	NR	NR	1		
Yamanaka et al. [35]	Cohort study	Japan/ 1980–1998	NR	LH: 11 (54.5)	60 ± 11	NR	NR	NR	100	OS, HR, 0.53 95% CI, 0.02–15.24; R0, NR
				RH: 14 (64.3)	55 ± 10	NR	NR	NR	93	

^a^Sign indicates median (range); otherwise, data are expressed as mean ± SD

^b^In addition to EBD and PBD, biliary drainage also includes EBD + PBD

*Abbreviations*: *NR* not reported in the text, *EBD* endoscopic biliary drainage, *PBD* percutaneous biliary drainage, *PVE* portal venous embolization

After quality evaluation, the scores of our included studies ranged from 6 to 8, based on the Newcastle–Ottawa Scale. As shown in the Table 2, there were 3 literatures with < 7 points and 8 literatures with ≥ 7 points.

**Table 2:** The quality evaluation of cohort studies

	Methodological quality			
Study	Selection	Comparability	Outcome	Quality score
Bednarsch et al. [28]	★★★★	★	★★	7 stars
Govil et al. [19]	★★★★	★	★	6 stars
Hong et al. [29]	★★★★	★★	★★	8 stars
Jo et al. [15]	★★★★	★★	★★	8 stars
Lee et al. [30]	★★★★	★	★★	7 stars
Otto et al. [31]	★★★★	★	★★	7 stars
Ratti et al. [32]	★★★★	★	★★	7 stars
Shimizu et al. [11]	★★★★	★	★★	7 stars
Sugiura et al. [33]	★★★★	★	★	6 stars
Konstadoulakis et al. [34]	★★★	★	★★	6 stars
Yamanaka et al. [35]	★★★★	★	★★	7 stars

### Primary outcomes: overall survival

**Fig. 2:**
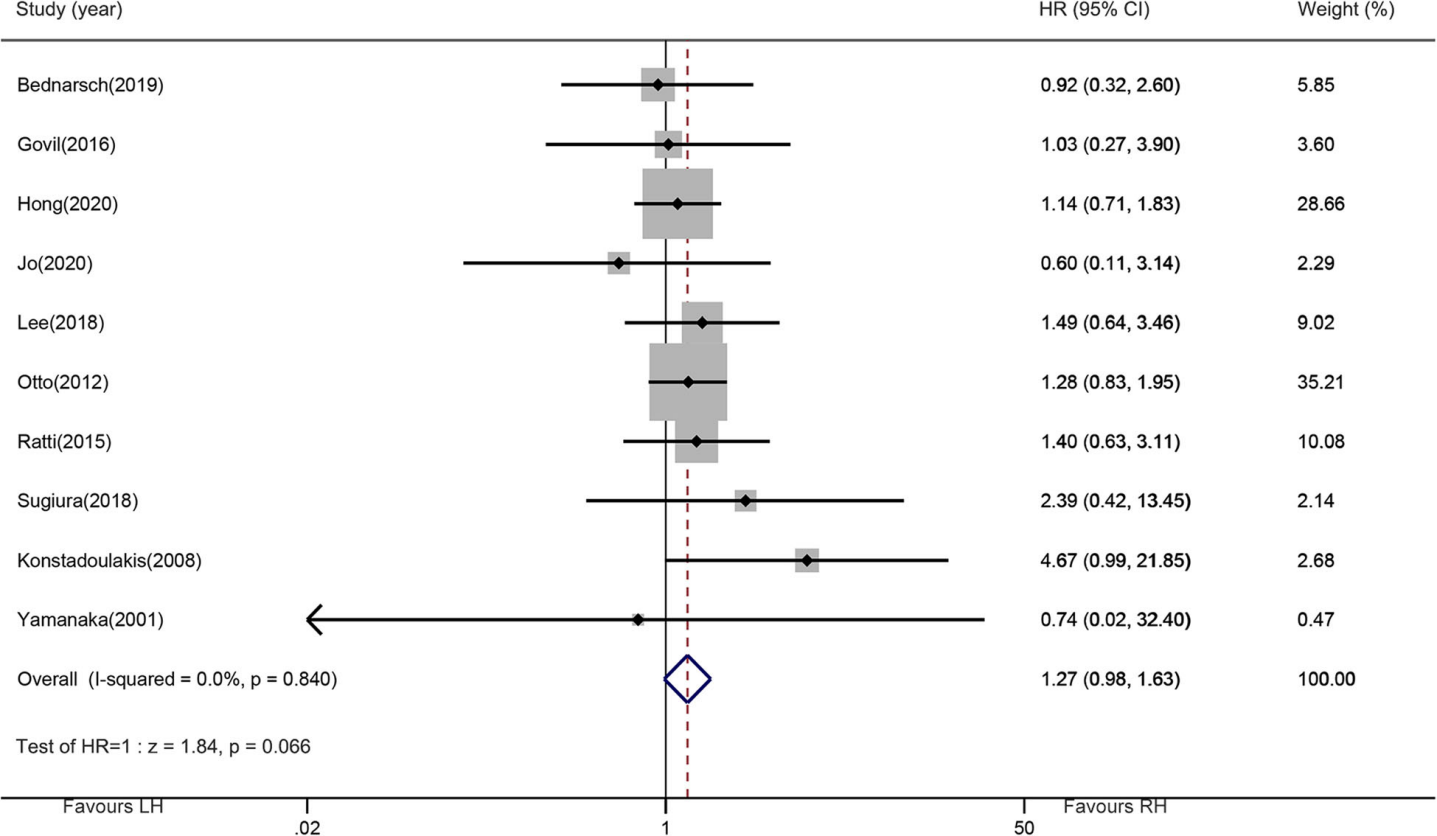
Forest plots of overall survival (left-side hepatectomy vs. right-side hepatectomy)

In order to evaluate the prognosis of patients under different surgeries, we analyzed the overall survival data from ten cohorts (including 859 patients) in this study, and the data were visualized by forest plots in Fig. 2. The pooled HR estimated based on the fixed-effects model and the random-effects model was 1.27 (95% CI, 0.98–1.63; *P* = 0.066), indicating that the difference between LH and RH was not statistically significant. No significant difference was observed among studies in the estimates for OS (I2 = 0%, *P*_heterogeneity_ = 0.840). Subgroup analysis showed that the analysis results did not change due to the region, year of publication, and the number of cases of left-side hepatectomy, and the differences between LH and RH remained not significant (Table 3).

**Table 3:** Subgroup analyses

Variable	Subgroup	OS	1-year survival	3-year survival	5-year survival	Overall morbidity	Major morbidity	PHLF	Mortality	R0 resection
Region	Western	HR = 1.34; 95% CI, 0.95–1.89; *P* = 0.097; *n* = 4	RR = 0.90; 95% CI, 0.72–1.13; *P* = 0.354 *n* = 2	RR = 0.93; 95% CI, 0.73–1.19; *P* = 0.552 *n* = 3	RR = 0.70; 95% CI, 0.52–0.94; *P* = 0.018 *n* = 4	RR = 0.70; 95% CI, 0.57–0.87; *P* = 0.001 *n* = 2	RR = 0.62; 95% CI, 0.42–0.91; *P* = 0.015 *n* = 2	RR = 0.40; 95% CI, 0.09–1.82; *P* = 0.233 *n* = 1	RR = 0.39; 95% CI, 0.17–0.88; *P* = 0.024 *n* = 3	RR = 0.87; 95% CI, 0.76–0.99; *P* = 0.038 *n* = 3
	Eastern	HR = 1.19; 95% CI, 0.82–1.73; *P* = 0.362; *n* = 6	RR = 1.08; 95% CI, 0.93–1.24; *P* = 0.315 *n* = 3	RR = 0.96; 95% CI, 0.77–1.19; *P* = 0.681 *n* = 4	RR = 0.97; 95% CI, 0.72–1.30; *P* = 0.836 *n* = 4	RR = 0.92; 95% CI, 0.74–1.14; *P* = 0.434 *n* = 2	RR = 0.88; 95% CI, 0.61–1.26; *P* = 0.477 *n* = 3	RR = 0.16; 95% CI, 0.05–0.55; *P* = 0.003 *n* = 3	RR = 0.42; 95% CI, 0.20–0.88; *P* = 0.021 *n* = 6	RR = 1.00; 95% CI, 0.90–1.10; *P* = 0.958 *n* = 4
Year of publication	≤2014	HR = 1.39; 95% CI, 0.92–2.10; *P* = 0.113; *n* = 3	RR = 0.90; 95% CI, 0.72–1.13; *P* = 0.354 *n* = 2	RR = 0.53; 95% CI, 0.29–0.96; *P* = 0.037 *n* = 1	RR = 0.63; 95% CI, 0.39–1.00; *P* = 0.051 *n* = 2	RR = 0.86; 95% CI, 0.61–1.20; *P* = 0.377 *n* = 1	*n* = 0	*n* = 0	RR = 0.32; 95% CI, 0.13–0.77; *P* = 0.011 *n* = 3	RR = 0.90; 95% CI, 0.78–1.04; *P* = 0.141 *n* = 2
	>2014	HR = 1.20; 95% CI, 0.87–1.65; *P* = 0.276; *n* = 7	RR = 1.08; 95% CI, 0.93–1.24; P = 0.315 *n* = 3	RR = 0.99; 95% CI, 0.84–1.18; *P* = 0.917 *n* = 6	RR = 0.89; 95% CI, 0.70–1.12; *P* = 0.307 *n* = 6	RR = 0.81; 95% CI, 0.68–0.97; *P* = 0.018 *n* = 3	*n* = 5	*n* = 4	RR = 0.47; 95% CI, 0.23–0.96; *P* = 0.038 *n* = 6	RR = 0.97; 95% CI, 0.88–1.07; *P* = 0.582 *n* = 5
Cases	> 41cases	HR = 1.24; 95% CI, 0.92–1.66; *P* = 0.154; *n* = 3	RR = 1.08; 95% CI, 0.93–1.25; *P* = 0.303 *n* = 2	RR = 0.93; 95% CI, 0.72–1.21; *P* = 0.594 *n* = 2	RR = 0.84; 95% CI, 0.62–1.13; *P* = 0.248 *n* = 3	RR = 0.85; 95% CI, 0.70–1.03; *P* = 0.102 *n* = 3	RR = 0.76; 95% CI, 0.46–1.27; *P* = 0.295 *n* = 2	RR = 0.38; 95% CI, 0.11–1.32; *P* = 0.127 *n* = 2	RR = 0.32; 95% CI, 0.14–0.72; *P* = 0.006 *n* = 4	RR = 0.95; 95% CI, 0.85–1.06; *P* = 0.369 *n* = 4
	≤ 41cases	HR = 1.35; 95% CI, 0.82–2.23; *P* = 0.231; *n* = 7	RR = 0.96; 95% CI, 0.77–1.19; *P* = 0.688 *n* = 3	RR = 0.95; 95% CI, 0.77–1.18; *P* = 0.656 *n* = 5	RR = 0.81; 95% CI, 0.61–1.08; *P* = 0.148 *n* = 5	RR = 0.74; 95% CI, 0.60–0.91; *P* = 0.004 *n* = 1	RR = 0.71; 95% CI, 0.52–0.97; *P* = 0.029 *n* = 3	RR = 0.12; 95% CI, 0.03–0.55; *P* = 0.006 *n* = 2	RR = 0.52; 95% CI, 0.24–1.10; *P* = 0.086 *n* = 5	RR = 0.94; 95% CI, 0.84–1.05; *P* = 0.289 *n* = 3

Data are presented as HR or RR (95% CI); *P* value; number of included studies (*n*)

*Abbreviations*: *OS* overall survival, *PHLF* postoperative liver failure

### Secondary outcomes

#### 1-, 3-, and 5-year survival rates

**Fig. 3:**
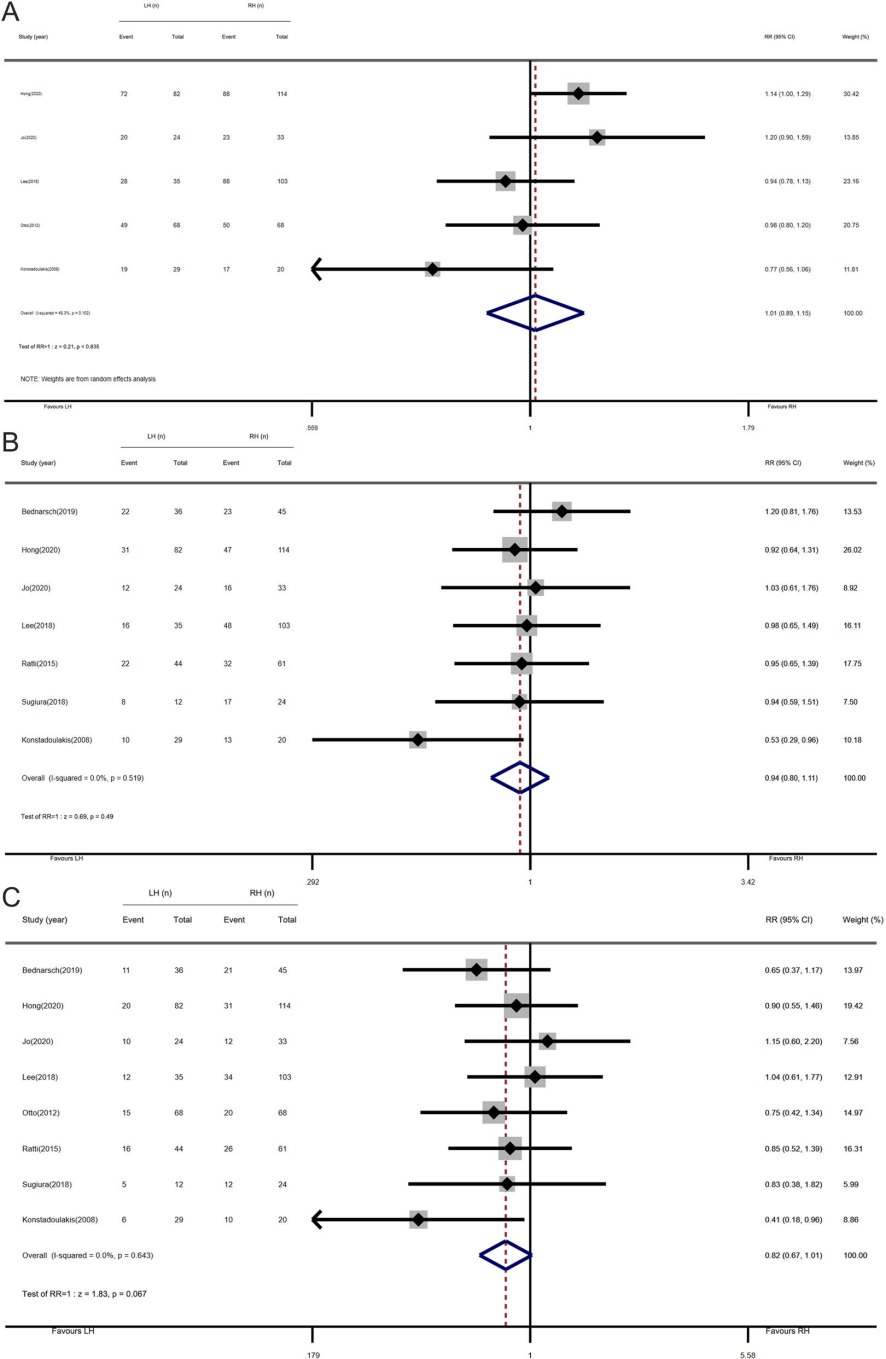
Forest plots of 1-, 3-, and 5-year survival rates (left-side hepatectomy vs. right-side hepatectomy). **a** 1-year survival rate. **b** 3-year survival rate. **c** 5-year survival rate

Five studies included 1-year survival data containing 576 patients, of which 79.0% (188 of 238) were in the LH group and 78.7% (266 of 338) in the RH group, seven included 3-year survival data containing 662 patients, of which 46.2% (121 of 262) were in the LH group and 49.0% (196 of 400) in the RH group; and eight included 5-year survival data containing 798 patients, of which 28.8% (95 of 330) were in the LH group and 35.5% (166 of 468) in the RH group. The results of the pooled 1-year, 3-year, and 5-year survival rates for LH vs. RH are shown in Fig. 3. Based on the random-effects model, the pooled RR for the 1-year survival rates was 1.01 (95% CI, 0.89–1.15; *P* = 0.835); the pooled RR for the 3- and 5-year survival rates calculated using the fixed-effects model was 0.94 (95% CI, 0.80–1.11; *P* = 0.49) and 0.82 (95% CI, 0.67–1.01; *P* = 0.067), respectively.

The results indicated that there was no statistically significant difference in the 1-, 3-, and 5-year survival rates between LH and RH. All studies on 1-year survival had no obvious heterogeneity (*I*^2^ = 48.3%, *P*_heterogeneity_ = 0.102). No statistically significant heterogeneity was observed for all studies on 3-year and 5-year survival rates (*I*^2^ = 0%, *P*_heterogeneity_ = 0.519; *I*^2^ = 0%, *P*_heterogeneity_ = 0.643, respectively).

Subgroup analysis demonstrated that despite the different publication years and the number of cases of left-side hepatectomy, the results of 1-, 3-, and 5-year survival rate showed no obvious difference. The 1- and 3-year survival rates under the subgroup of different regions were also the same. However, patients who underwent LH in western centers were associated with poor 5-year survival results (Table 3).

### Overall postoperative morbidity and major postoperative morbidity

**Fig. 4:**
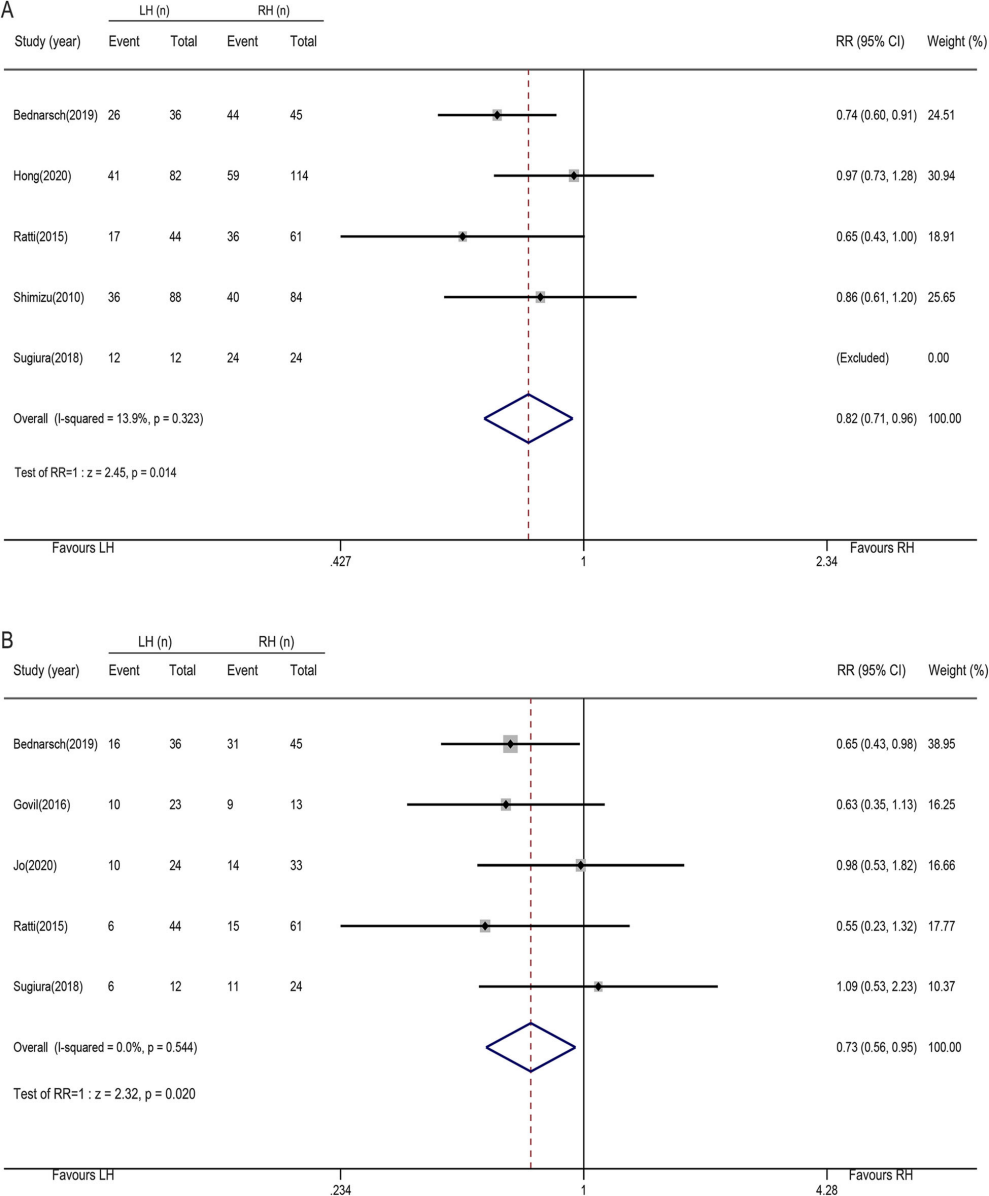
Forest plots of overall postoperative morbidity and major postoperative morbidity (left-side hepatectomy vs. right-side hepatectomy). **a** Overall postoperative morbidity. **b** Major postoperative morbidity

Five studies with 590 patients provide information on overall postoperative morbidity, with rates of 50.4% (132 of 262) in the LH group and 61.9% (203 of 328) in the RH group. As shown in Fig. 4a, the pooled RR was 0.82 (95% CI, 0.71–0.96; *P* = 0.014), and the overall morbidity of the LH group was significantly lower than that of the RH group. The heterogeneity between the studies was not obvious (*I*^2^ = 13.9%, *P*_heterogeneity_ = 0.323). Major postoperative morbidity was mentioned in five studies with 315 patients; major morbidity occurred in 34.5% (48 of 139) of patients in the LH group and 45.5% (80 of 176) in the RH group. The pooled RR was 0.73 (95% CI, 0.56–0.95; *P* = 0.020; Fig. 4b). The results suggested that RH group had a higher risk of serious postoperative complications, and there was no heterogeneity among the studies (*I*^2^= 0%, *P*_heterogeneity_= 0.544).

Subgroup analysis indicated that LH was associated with reduced overall morbidity in post-2014, western center studies and less-experienced centers (≤ 41cases). However, in eastern center and pre-2014 studies, there was no relationship between the two procedures and overall morbidity. All major morbidity data were collected from the studies published after 2014. The results of the western center studies and less-experienced centers were consistent with the meta-analysis, but no significant differences were observed in the eastern center studies (Table 3).

### Post-hepatectomy liver failure and procedure-related mortality

**Fig. 5:**
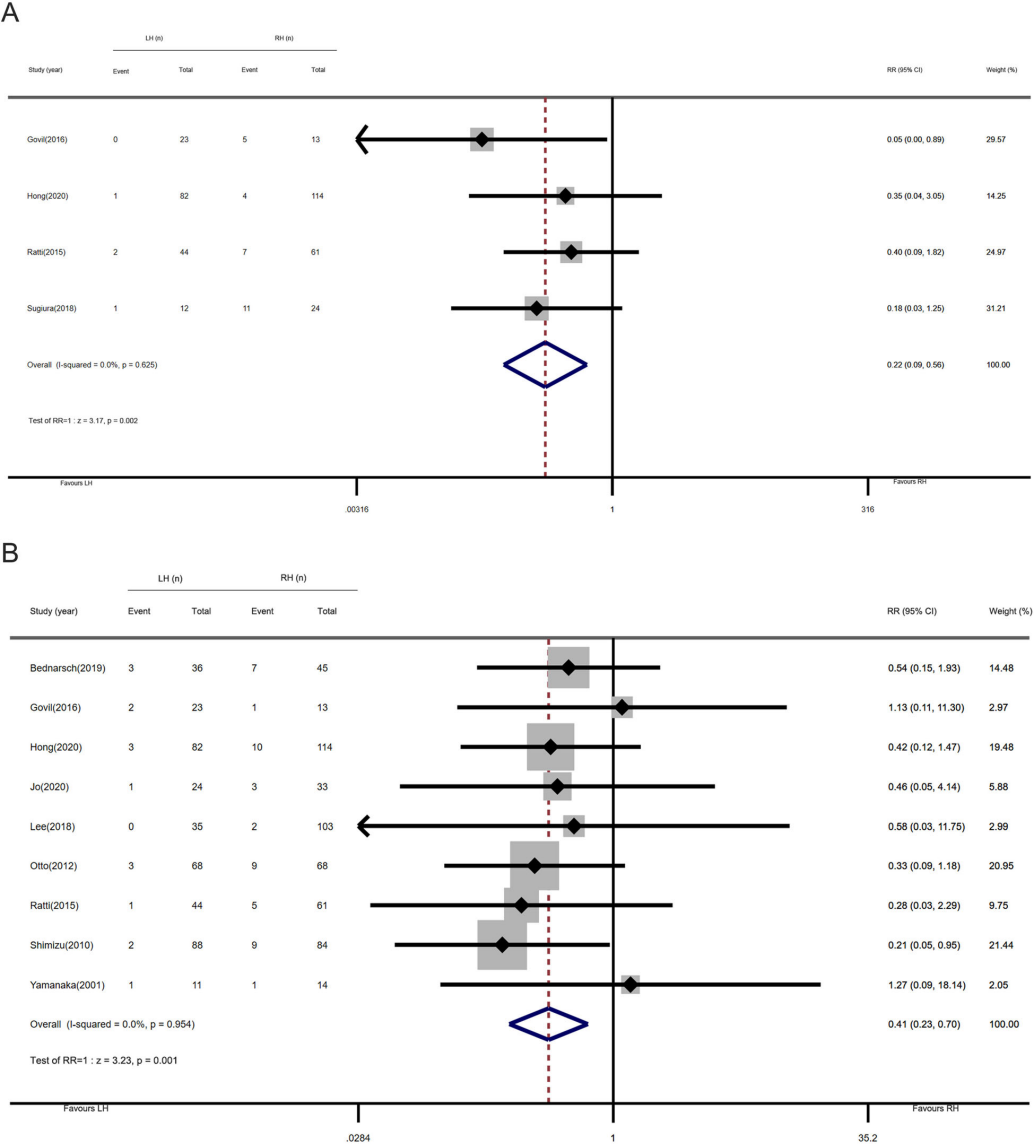
Forest plots of post-hepatectomy liver failure and procedure-related mortality (left-side hepatectomy vs. right-side hepatectomy). **a** Post-hepatectomy liver failure. **b** Procedure-related mortality

Four studies reported data about post-hepatectomy liver failure in 373 patients. In the LH and RH group, PHLF rate was 2.5% (4 of 161) and 12.7% (27 of 212), respectively. Figure 5a shows the pooled results of the fixed-effects model; the pooled RR for PHLF was 0.22 (95% CI, 0.09–0.56; *P* = 0.002). These results showed that performing LH could reduce the possibility of post-hepatectomy liver failure. Nine studies with 976 patients reported perioperative mortality. The mortality rates in the LH group and RH group were 3.9% (16 of 411) and 8.8% (47 of 535), respectively. As depicted in the forest plots, the pooled RR was 0.41 (95% CI, 0.23–0.70; *P* = 0.001), and LH significantly reduces perioperative mortality relative to RH (Fig. 5b). For post-hepatectomy liver failure and postoperative mortality, no heterogeneity was observed between different studies (*I*^2^ = 0%, *P*_heterogeneity_ = 0.625; *I*^2^= 0%, *P*_heterogeneity_ = 0.954, respectively).

In the subgroup analysis, the results of the eastern center and less-experienced centers showed that the LH group presented a lower incidence of PHLF. Regarding mortality, regardless of changes in region and publication year, LH was significantly associated with lower mortality. And in centers where LH was performed in more than 41 cases, the mortality rate was lower (Table 3).

### R0 resection rate

**Fig. 6:**
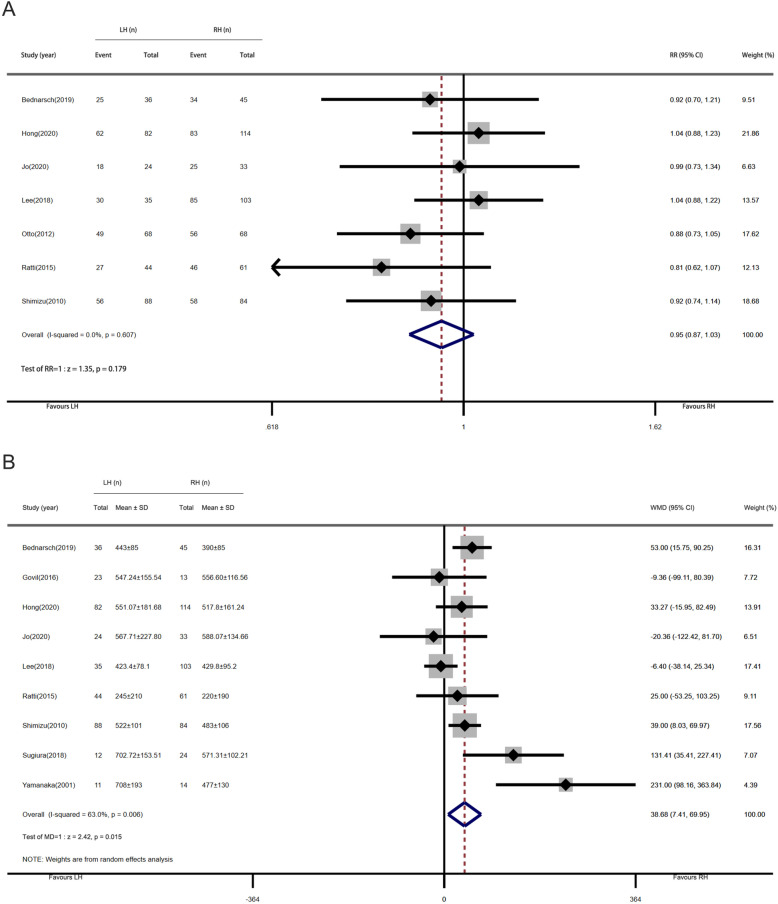
Forest plots of R0 resection rate and operating time (left-side hepatectomy vs. right-side hepatectomy). **a** R0 resection rate. **b** Operating time

A total of 7 studies reported R0 resection rate of 885 HCCA patients. In the LH group, 70.8% (267 of 377) of patients achieved negative margin, while in the RH group, the data was 76.2% (387 of 508). The pooled analysis results showed that the RR of R0 resection rate was 0.95 (95% CI, 0.87–1.03; *P* = 0.179) without heterogeneity (*I*^2^ = 0%, *P*_heterogeneity_ = 0.607; Fig. 6a). No statistical difference in R0 resection rate between LH and RH was identified. Subgroup analysis showed that the results of the western center were inconsistent with the meta-analysis, that is, a higher R0 resection rate could be obtained by RH (Table 3).

### Operation time

Nine studies with a total of 846 patients reported operating time. Based on the fixed-effects model, there was a low level of heterogeneity between the studies (*I*^2^ = 45.1%, *P*_heterogeneity_ = 0.078). Considering *I*^2^ as critical at 50%, the random-effects model was used to pool the studies in a more conservative way. As shown in Fig. 6b, the pooled MD was 38.68 (95% CI, 7.41–69.95; *P* = 0.015), indicating that the operation time in the LH group was significantly longer than that in the RH group.

### Publication bias

**Fig. 7:**
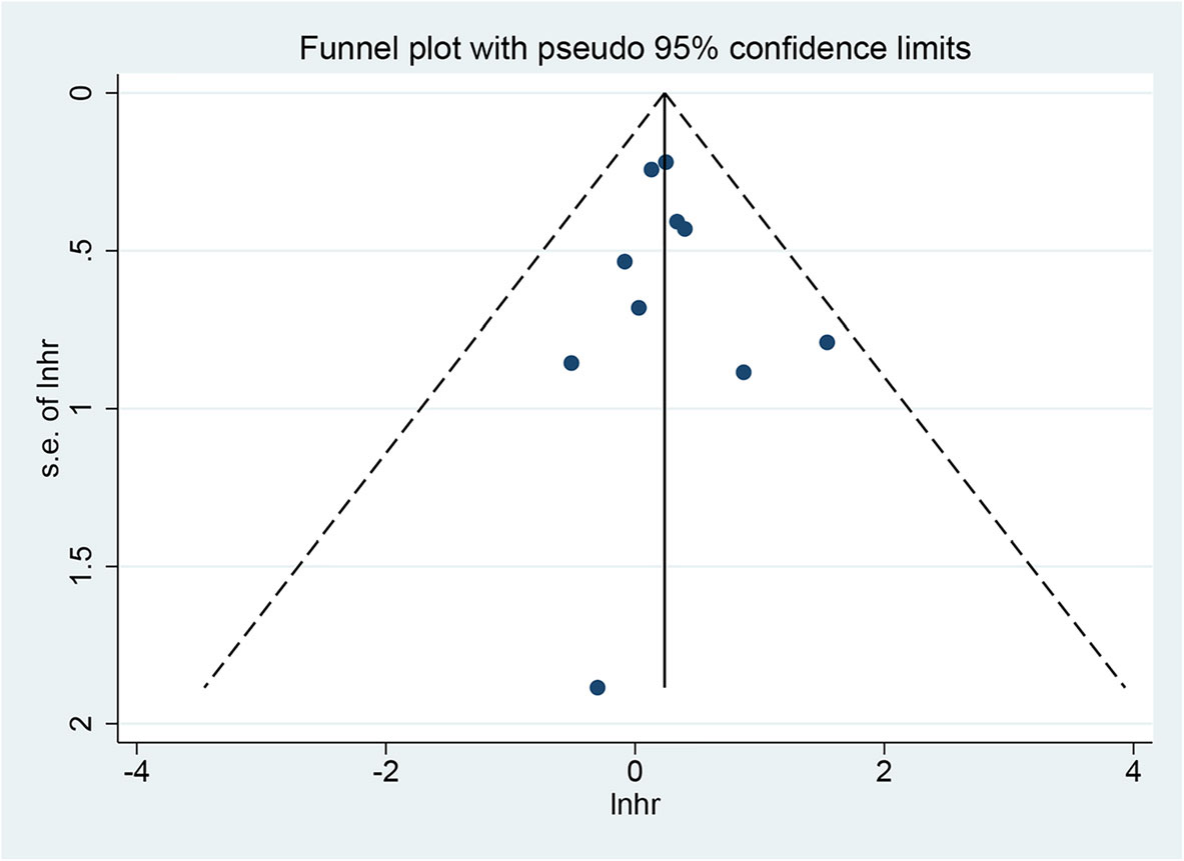
Funnel plot of overall survival

Figure 7 shows a funnel plot of OS. Neither Begg’s test nor Egger’s test found significant publication bias, that is, the *P* values for the outcome was greater than 0.05. Since the number of studies included in other endpoints in the meta-analysis was small, funnel plots, Begg’s test, and Egger’s test were not performed to assess publication bias.

## Discussion

The evidence indicated that the effect of palliative treatment for HCCA was limited, and surgery is the only treatment that can improve long-term survival. Bile duct resection combined with major hepatectomy has been regarded as the standard surgical method for HCCA. In order to compare the efficacy and safety of LH and RH, we performed this meta-analysis. The results of our analysis show that LH is comparable to RH in terms of long-term survival. However, comparing with RH, LH has reduced overall morbidity, major morbidity, postoperative liver failure, mortality rates, and longer operative time. Furthermore, it has been found that no significant difference existed in the rate of R0 resection between LH and RH.

It is common that radical surgery with negative margins is the only effective treatment for HCCA. Therefore, it is significant to identify the opportunities of surgical treatment for HCCA patients. At the same time, preoperative imaging plays a key role in determining the type of operation. These all require precise preoperative diagnosis, tumor staging, tumor localization, and evaluation of FLR. Preoperative diagnosis methods include abdominal ultrasound, multi-detector-row computed tomography (MDCT), magnetic resonance imaging (MRI), positron emission tomography/computed tomography (PET/CT), and invasive examinations such as laparoscopy and cholangiography. A number of studies have shown that MDCT is more accurate in evaluating biliary and vascular involvement [36], and a research by Fukami et al. has also proved that multi-slice spiral CT is useful for evaluating right hepatic artery (RHA) invasion of perihilar cholangiocarcinoma [37]. Additionally, this conclusion is of great significance to the formulation of the surgical plan for HCCA that mainly involves the left side and RHA, that is, LH plus RHA resection and reconstruction. MRI is considered to be equivalent to MDCT and can be used as an imaging technique to replace MDCT [38]. Furthermore, MRI as well as MDCT plays an important role in calculating FLR [39]. As for the judgment of lymph node metastasis, MDCT has limited accuracy [36, 38], while PET/CT performs better in judging lymph node metastasis, with a sensitivity of 67.9% and a specificity of 88.0%. And it performs better in assessing the liver, peritoneum, or other distant metastasis [40]. However, it is difficult for PET/CT to distinguish benign and malignant lesions [41]. In a word, every preoperative imaging method has their own advantages, and for each specific patient, an optimized and individualized preoperative examination strategy should be developed to provide guidance for treatment.

Due to technical limitations and anatomical disadvantages, many surgeons choose RH [10, 12]. But recently, more centers began to take LH into the HCCA clinical treatment. The present study showed that the long-term survival of LH is not worse than that of RH. Subgroup analysis demonstrated that only the western center group performed better on 5-year survival after RH. The results of the eastern center group, the different publication years, and different cases of LH were analyzed in a meta-analysis. Some authors thought that R0 resection was the most important factor for improving survival after surgical resection [4, 42]. Here, in our meta-analysis including a subgroup analysis, the results were consistent, suggesting that among HCCA patients treated with surgery, R0 resection may be the most important factor for improving survival. The present results provided convincing evidence that there is a positive effect of R0 resection on long-term survival. Therefore, it is reasonable to assume that in addition to tumor location, R0 resection rate is also a determinant of surgical procedure.

Neuhaus et al. [43] recommended additional caudate lobectomy to increase radicality, while more supporters believed that caudate lobectomy should be performed routinely based on anatomical and histopathological perspectives [44, 45]. Birgin et al. [46] presented a pooled RR value of 1.40, which was based on the 4 studies, reflecting a higher risk of residual tumors at the resection margin in patients without the caudate lobectomy. In the studies that we included, all the patients have undergone caudate lobe resection, but in some studies, only 80–90% patients have undergone caudate lobe resection. In view of partial incomplete data and unclear implementation criteria, the subgroup analysis based on caudate lobectomy was regrettably not performed.

In a matched cohort study of Hosokawa et al., the long-term survival, short-term outcomes, and R0 resection of left trisectionectomy (LT) and right hemihepatectomy were comparable [47]. In Esaki et al.’s study, the three groups of left trisectionectomy, left hemihepatectomy, and right hemihepatectomy were compared. The results showed that although the Grade IIIa complications of the LT group were higher than those of the other two groups, the survival of the three groups was comparable. And there was no difference in R0 and overall morbidity between the LT group and right hemihepatectomy group [48]. Natsume et al. focused on the comparison of clinical significance between LT and left hemihepatectomy indicating that the overall morbidity of LT was significantly higher than that of left hemihepatectomy, but the mortality and 5-year survival rates of the two groups were similar [10].

Analysis of morbidity and mortality revealed that LH was associated with better short-term outcomes, with both overall and major morbidity, postoperative liver failure, and mortality. A subgroup analysis also confirms this result. Of note, the overall morbidity of the LH group is only lower in studies published after 2014 and in western centers. As for major morbidity, the LH group performed better than the RH group in the subgroup analysis of region (in the western center). The differences in the eastern and western treatment strategies could be further investigated to provide ideas for further optimization of surgery. It was interesting that the PHLF rate of the eastern center group after performing LH was significantly reduced which was considered to be related to the active biliary drainage and PVE in the eastern center group [49]. However, there is only one study in the western center group in the subgroup analysis, so the results should be interpreted with cautiously caution. In addition, the date of major morbidity and PHLF were provided by studies published after 2014. It was not clear whether advances in technology and perioperative management in recent years have improved major morbidity and PHLF. When reporting mortality, the criteria for counted days were not uniformed. In theory, 30 days had a reduced mortality rate compared to 90 days, and due to the inconsistent days, it may post misleading influence on our final results. In the subgroup analysis with different numbers of LH cases, in the more experienced centers, the mortality rate of the LH group was lower than that of the RH group, which is also consistent with our conventional understanding. However, for the mortality rate including PHLF, the centers with the number of examples < 41 cases can obtain better results in the LH group. We consider that these centers have less experience, so they will be more cautious in surgery and have a more detailed surgery management, but the final mortality rate may still be related to experience. Since the number of studies included in the meta-analysis is not sufficient, the understanding of the results needs to be more cautious, and we hope that there will be more related study to be included in the future.

The PHLF caused by insufficient residual liver volume after major hepatectomy is the most fatal complication, with a mortality rate of 52–68% [50]. Kawasaki et al. [12]. showed that patients with HCCA routinely performed biliary drainage and PVE before extensive hepatectomy, and the hospital mortality rate can be reduced to as low as 1.3%. Preoperative drainage is thought to improve liver function in patients with jaundice, which could reduce PHLF and death. Endoscopic biliary stenting and endoscopic nasobiliary drainage are superior to percutaneous transhepatic biliary drainage because they can reduce the incidence of tumor spread. And given the increase in major morbidity, routine preoperative drainage is not recommended [51]. PVE is believed to increase FLR, but there is no consensus on the indication criteria. In this meta-analysis, various studies conducted biliary drainage and PVE under the premise of different standards, hoping to further clarify the indications of biliary drainage and PVE in the future.

This meta-analysis has some limitations. Firstly, due to the rarity of HCCA, the included studies were all cohort studies, and there are no randomized controlled trials, which would cause selection bias. The quantity of sample is insufficient in some studies, and differences in treatment experience may affect the accuracy of the results. Secondly, there was heterogeneity among the studies on 1-year survival rate and operation time, but the degree was low. Thirdly, the Bismuth classification of tumors in each study is also different, but the data is not sufficient for subgroup analysis based on Bismuth classification. We have taken specific records of every surgery including hemihepatectomy, extended hemihepatectomy, and trisectionectomy. However, samples in most studies were not sufficient to conduct further analysis of surgery because of low incidence of HCCA. It is hoped that further analysis based on different Bismuth classification and treatment modality would be conducted in the future.

In conclusion, the present meta-analysis suggests that for resectable HCCA patients, LH and RH have comparable survival benefits, R0 resection rates, and lower morbidity and mortality. LH is safe and feasible. We recommend that the choice of LH or RH should be based on the specific anatomy of the tumor to achieve radical cure as much as possible, while optimizing perioperative management to reduce postoperative morbidity and mortality.

To the best of our knowledge, this is the first meta-analysis comparing the outcomes of LH and RH to date. Moreover, detailed data about preoperative drainage and PVE were analyzed. We found that although former studies had performed well in comparing LH and RH for HCCA, there still remained a number of aspects to be improved, such as Bismuth Clarification, preoperative drainage, preoperative portal vein embolization, and vascular resection. We hope more comprehensive and detailed data about these aspects to be provided in the following researches, and if so, the more convinced results of meta-analysis can be concluded for clinical treatments. And given the low incidence of HCCA, further randomized trials in the real-world may also be needed.

## Supplementary Information

**Additional file 1 Supplementary Table 1.** Search strategy.

## Data Availability

All data are fully available without restriction.

## Abbreviations

HCCA: Hilar cholangiocarcinoma LH Left-side hepatectomy RH Right-side hepatectomy OS Overall survival HR Hazard ratio RR Relative risk MD Mean difference CI Confidence interval FLR Future liver remnant PHLF Postoperative liver failure PVE Portal vein embolization PRISMA Preferred Reporting Items for Systematic Review and Meta-analyses MOOSE Meta-analysis of Observational Studies in Epidemiology RCTs Randomized controlled trials PBD Percutaneous biliary drainage EBD Endoscopic biliary drainage NOS Newcastle–Ottawa quality assessment scale SD Standard deviation
