# Supplementary material for: Left-side vs. right-side hepatectomy for hilar cholangiocarcinoma: a meta-analysis
Source: World J Surg Oncol. 2021 Apr 10;19:107. doi: 10.1186/s12957-021-02213-6 (PMC8037893; doi:10.1186/s12957-021-02213-6)
Supplement: Supplementary file 1 — Additional file 1 Supplementary Table 1. Search strategy. [file 12957_2021_2213_MOESM1_ESM.docx]

**Supplementary Table 1.** Search strategy

| **Database** | **Search keywords** |
| --- | --- |
| PUBMED | **MEDLINE**   1. Klatskin Tumor [MeSH Terms] 2. Tumor, Klatskin OR Klatskin's Tumor OR Klatskins Tumor OR Tumor, Klatskin's OR Hilar Cholangiocarcinoma OR Cholangiocarcinoma, Hilar OR Cholangiocarcinomas, Hilar OR Hilar Cholangiocarcinomas OR Perihilar Cholangiocarcinoma OR Cholangiocarcinoma, Perihilar OR Cholangiocarcinomas, Perihilar OR Perihilar Cholangiocarcinomas 3. #1 or #2 4. Hepatectomy [MeSH Terms] 5. (liver OR hepatic) AND (surg* OR resect* OR segmentect* OR hepatect*) 6. #4 OR #5 7. ((Left-Side) OR (Left)) AND ((Right-side) OR (Right)) 8. #6 AND #7 9. (RH OR RLR) AND (LH OR LLR) 10. #8 OR #9 11. #10 AND #3   **Limitation:** humans  **Date of Search:** March 02, 2020  **Results:** 234 articles were found |
| EMBASE | **EMBASE**   1. ‘klatskin tumor’/exp OR ‘klatskin tumor’/syn OR ‘hcca’ OR ‘pcca’ 2. (‘hilar’ OR ‘perihilar’) AND (‘Cholangiocarcinoma’ OR ‘Cholangiocarcinomas’) 3. #1 OR #2 4. ‘liver resection’/exp AND ‘liver resection’/syn 5. ‘hepatic’ AND (‘surgery’ OR ‘resection’ OR ‘segmentectomy’ OR ‘hepatectomy’) 6. #4 OR #5 7. (‘left-side’ OR ‘left’) AND (‘right-side’ OR ‘right’) 8. #6 AND #7 9. (‘rh’ OR ‘rlr’) AND (‘lh’ OR ‘llr’) 10. #8 OR #9 11. #3 AND #10 12. [humans]/lim 13. #11 AND #12   exp = explosion search, syn = synonymous search, lim = limitation  **Limitation:** humans  **Date of Search:** March 02, 2020  **Results:** 455 articles were found |
| COCHRANE | **CENTRAL database (The Cochrane Library)**   1. MeSH descriptor: [Klatskin Tumor] explode all trees 2. Tumor, Klatskin OR Klatskin's Tumor OR Klatskins Tumor OR Tumor, Klatskin's OR Hilar Cholangiocarcinoma OR Cholangiocarcinoma, Hilar OR Cholangiocarcinomas, Hilar OR Hilar Cholangiocarcinomas OR Perihilar Cholangiocarcinoma OR Cholangiocarcinoma, Perihilar OR Cholangiocarcinomas, Perihilar OR Perihilar Cholangiocarcinomas 3. #1 OR #2 4. MeSH descriptor: [Hepatectomy] explode all trees 5. ((liver) OR (hepatic)) AND ((surg*) OR (resect*) OR (segmentect*) OR   (hepatect*))   1. #4 OR #5 2. ((Left-Side) OR (Left)) AND ((Right-side) OR (Right)) 3. #6 AND #7 4. (RH OR RLR) AND (LH OR LLR) 5. #8 OR #9 6. #3 AND #10   **Limitation:** none  **Date of Search:** March 02, 2020  **Results:** 3 articles were found |
